# Supplementary material for: Spike-timing-dependent plasticity learning of coincidence detection with passively integrated memristive circuits
Source: Nat Commun. 2018 Dec 14;9:5311. doi: 10.1038/s41467-018-07757-y (PMC6294012; doi:10.1038/s41467-018-07757-y)
Supplement: Supplementary file 1 — Supplementary Information [file 41467_2018_7757_MOESM1_ESM.pdf]

Supplementary Information

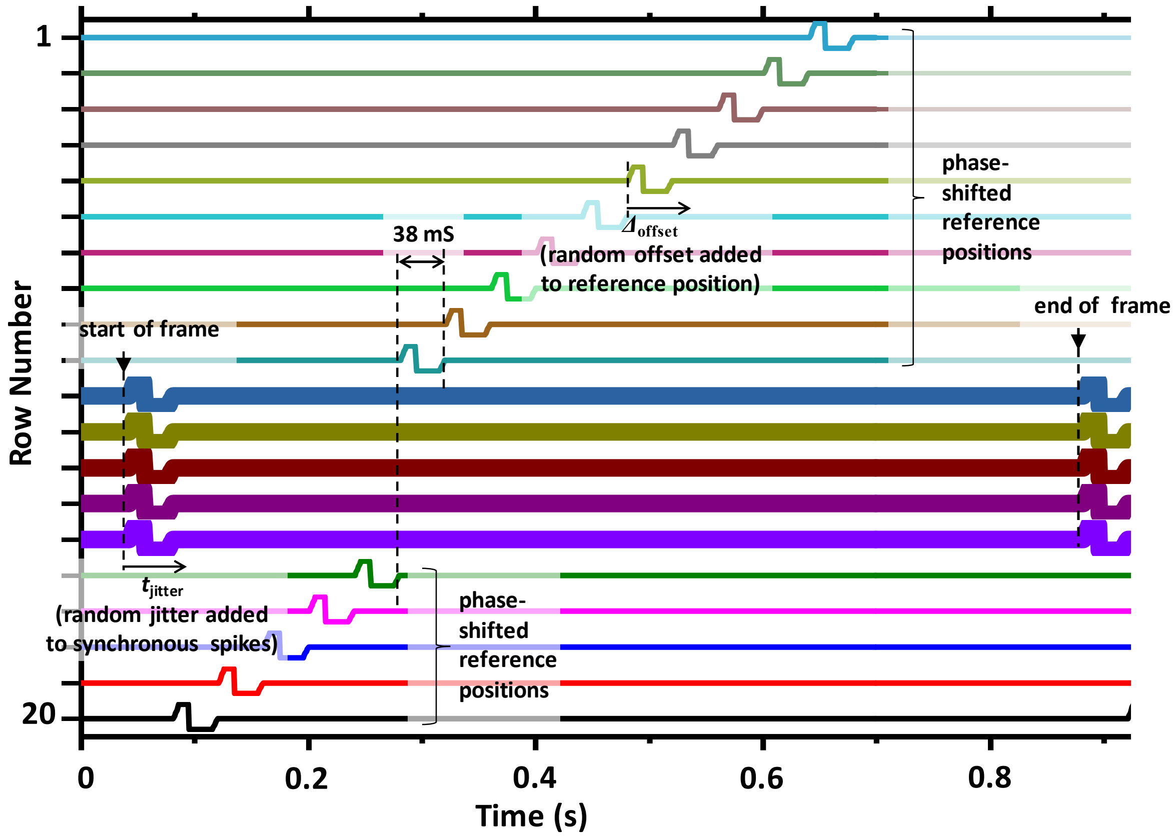

Supplementary Figure 1. Spike generation scheme. Key parameters for spike generation algorithm. (Similar to Figs. 2a and 3a-d synchronous spikes are shown with thicker lines.) The durations of all spikes, including rise and fall times, are always 38 ms. The reference positions of synchronous spikes are always at the start of frame. The references for noise spikes are staggered in time without a gap, starting with the spike at the highest row number. t_{jitter} and Δ_{offset} are random variables drawn from uniform distributions $[-(t_{jitter})_{max}, +(t_{jitter})_{max}]$ and $[-(\Delta_{offset})_{max}, +(\Delta_{offset})_{max}]$. If spike position moves outside of the frame after adding jitter or offset to the reference position, this spike is generated in the corresponding adjacent frame. Note that there is an additional 200 ms between the reference position of the last spike and the end of frame.

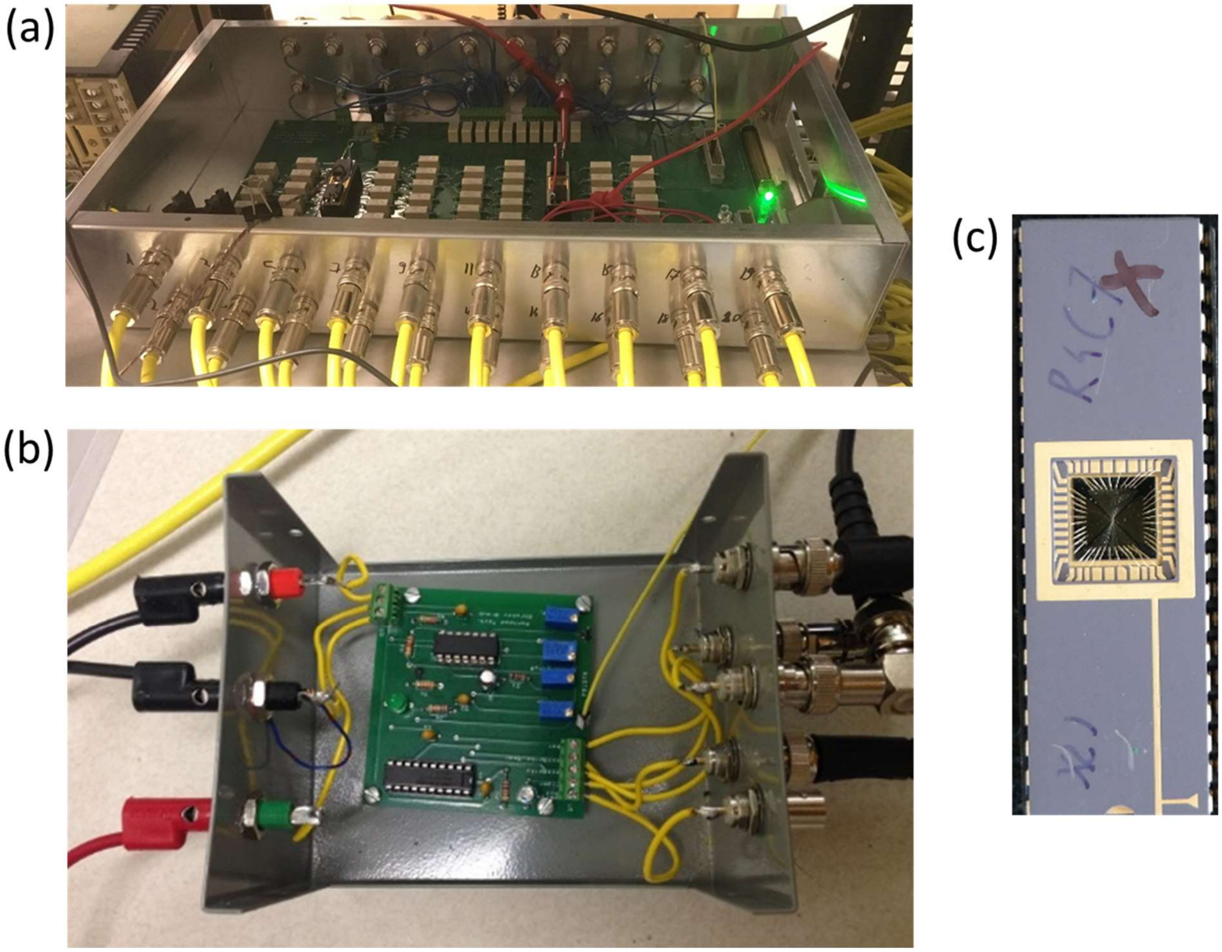

Supplementary Figure 2. Photos of experimental setup. (a) Printed circuit board hosting switching matrix, based on low-noise low-leakage mechanical relays, and memristive crossbar arrays. (b) Leaky-integrate-and-fire neuron implementation with discrete integrated circuit components of the circuit shown in Figure 2c. (c) Packaged memristive crossbar circuit. The detailed discussion of PCB functionality and the typical neuron circuit parameters are provided in Methods section.
